# Case Report: Atypical posttraumatic fat embolism syndrome: a case of rapidly progressive respiratory failure precipitated by a finger crush injury

**DOI:** 10.3389/fmed.2026.1950746

**Published:** 2026-09-22

**Authors:** Qingfa Liu, Tingting Wei

**Affiliations:** Department of Respiratory Medicine, Liaocheng People’s Hospital, Liaocheng, Shandong, China

**Keywords:** bronchoalveolar lavage, corticosteroids, fat embolism syndrome, finger crush injury, obesity

## Abstract

**Background:**

Fat embolism syndrome (FES) is classically associated with long bone fractures; however, digital crush injuries are an extremely rare precipitating factor. Obesity has been identified as an independent risk factor for the development of FES. The potential for rapid disease progression within 12 h of injury necessitates a high index of clinical suspicion.

**Case summary:**

A 43-year-old obese male underwent surgical debridement for crush injuries to his left index and thumb fingers. Twelve hours postoperatively, he developed exertional chest tightness. As the disease progressed, escalation from non-invasive to invasive mechanical ventilation became necessary. FES was confirmed by the detection of floating fat droplets and Oil Red O-positive lipid-laden macrophages in bronchoalveolar lavage fluid. After corticosteroid and fluid therapy, pulmonary infiltrates improved markedly, and the patient was successfully weaned from ventilation and extubated on day 7.

**Conclusions:**

This case confirms the diagnostic utility of bronchoalveolar lavage in conjunction with Oil Red O staining for rapid FES diagnosis, broadens the etiological spectrum to include atypical injuries of the short bones, and underscores the necessity of maintaining a high index of suspicion for FES in obese patients presenting with unexplained respiratory failure following finger crush injuries, thereby highlighting that early recognition and timely intervention are critical for improving prognosis.

## Introduction

Fat Embolism Syndrome is a systemic disorder primarily triggered by the entry of fat globules infat embolism syndrome, finger crush injury, obesity, bronchoalveolar lavage, corticosteroids to the circulatory system following long bone fractures or pelvic trauma (1). Its clinical manifestations are highly variable, with predominant involvement of the respiratory, central nervous, and integumentary systems (1). Epidemiological estimates indicate an overall incidence ranging from 0.04% to 0.12%, increasing to 1.29% among individuals under 30 years of age, as well as in those with closed femoral fractures or multiple long bone fractures (2). High-risk populations include males and patients sustaining multiple femoral fractures (3). Prognosis is generally poor, with severe cases potentially leading to respiratory failure, neurological deficits, and death (4, 5).

The diagnosis of FES, however, presents significant challenges due to the non-specific nature of its early symptoms, which often mimic acute emergencies such as pulmonary embolism. Moreover, existing diagnostic criteria are largely based on clinical observation rather than objective quantitative metrics. In clinical practice, chest CT scans and Oil Red O staining of bronchoalveolar lavage fluid are commonly employed diagnostic tools. Concerning treatment, current protocols emphasize supportive care, while the role of corticosteroids remains a subject of debate (6). Some case reports suggest that corticosteroids may facilitate lesion resolution (7). However, such evidence is derived primarily from isolated case reports or small-scale studies and lacks validation from robust randomized controlled trials sufficient to justify routine administration. Prompt diagnosis is critical for improving prognosis, as delayed recognition may delay the initiation of targeted interventions, thereby increasing mortality.

This article presents a case of a 43-year-old obese male who rapidly developed FES symptoms within 12 h postoperatively, triggered by an atypical factor: a finger crush injury, and progressed from non-invasive to invasive mechanical ventilation within the subsequent 7 h. The patient presented without long bone fractures or other common high-risk factors, yet followed a typical and severe clinical course. This case underscores the importance of early recognition and timely intervention for FES following atypical trauma and urges clinicians to maintain a high index of suspicion for such rare triggers, thereby reducing missed diagnoses and improving outcomes.

## Case presentation

A 43-year-old man presented to the emergency department complaining of unrelenting chest tightness that had persisted for 12 h. His medical history was notable for facial paralysis that had lasted six months, and he had undergone surgical repair for a crush injury to his left hand just one day prior to admission (Figure 1A). After the operation, the patient began experiencing chest constriction that progressively intensified. While breathing ambient air, his peripheral oxygen saturation fell to 84%, accompanied by tachycardia with a heart rate of 123 beats per minute. Notably, he had no accompanying symptoms such as cough, sputum expectoration, fever, syncope, altered consciousness, or cutaneous rash. Immediate management included initiation of non-invasive ventilatory support, after which he was transferred to the respiratory intensive care unit.

**Figure 1:**
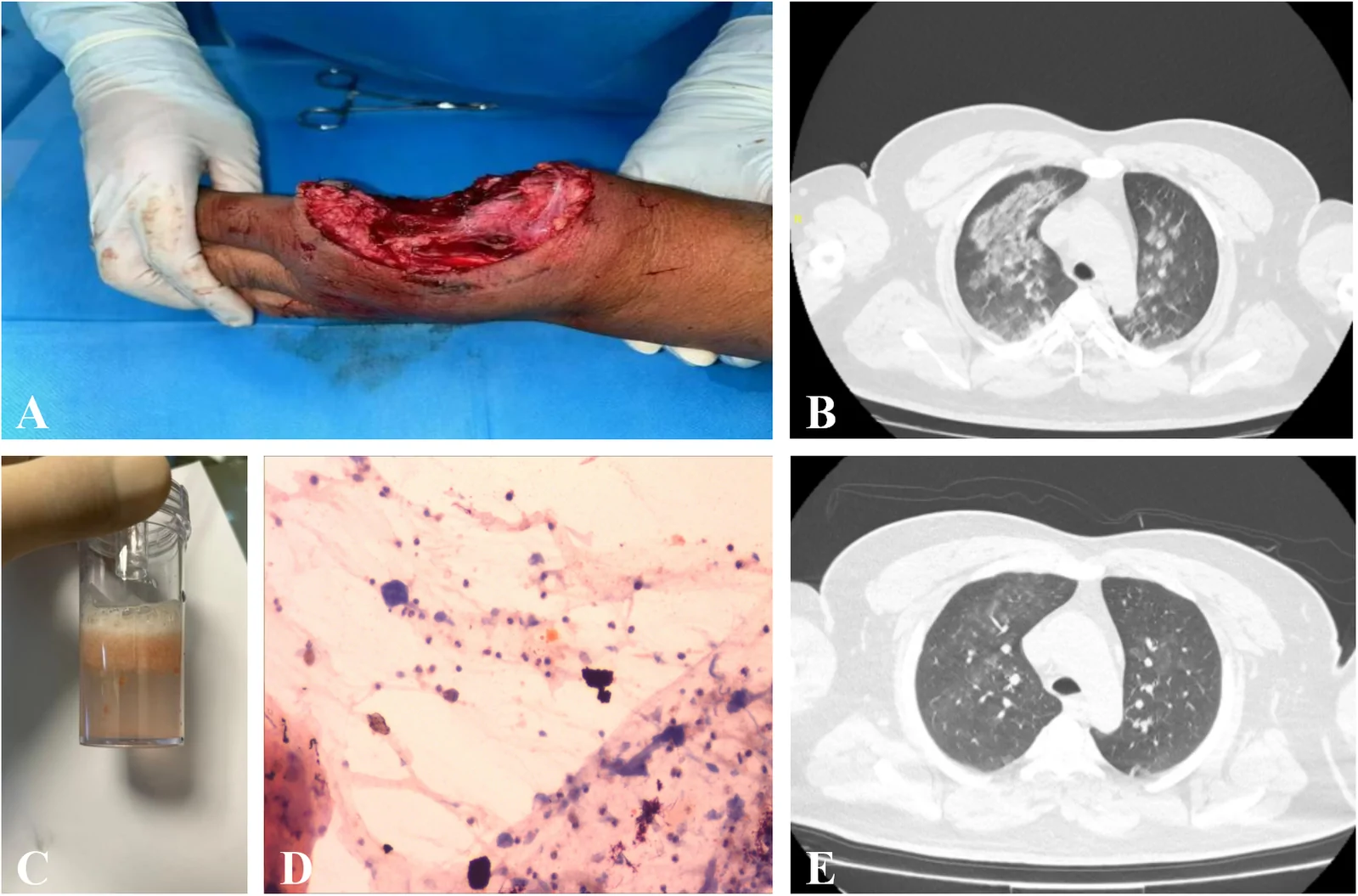
Signs of fat embolism. **(A)** Condition of the hand after debridement peration. **(B)** Chest CT upon admission. **(C)** Bronchoalveolar lavage fluid collected via bronchoscopy. **(D)** Pathological section of bronchoalveolar lavage fluid stained with Oil Red O. **(E)** Chest CT scan after 48 h of treatment.

Upon arrival at the respiratory intensive care unit, the patient's hemodynamic and respiratory parameters were recorded as follows: core temperature 36.5 °C, arterial pressure 146/74 mmHg, pulse 103 bpm, respiratory frequency 20 breaths/min, and peripheral oxygen saturation 95.8%, while the patient was receiving non-invasive ventilation with an FiO₂ of 50%. Anthropometric assessment revealed a height of 173 cm and a body mass of 135 kg. Physical examination identified a surgical dressing on the left hand, alongside a patent drainage catheter functioning without obstruction. Pulmonary auscultation demonstrated bilaterally diminished breath sounds accompanied by moist crackles, yet no wheezing or pleural friction rubs were detected. Cardiac evaluation showed a regular rhythm with normal heart sounds, no murmurs or friction rubs, and the lower extremities presented no edema.

The complete blood count revealed a white blood cell count of 10.53 × 10⁹/L, with neutrophils comprising 93.0% and hemoglobin measuring 158 g/L. High-sensitivity C-reactive protein was elevated to 26.2 mg/L, serum procalcitonin was 0.23 ng/mL, cardiac troponin I was within normal limits at 0.01 µg/L, NT-proBNP measured 327 pg/mL, D-dimer was 1.06 mg/L, and albumin was 40 g/L. Assessments of hepatic and renal function, viral serology, and cardiac troponin I levels were all unremarkable. Arterial blood gas analysis performed upon admission, under conditions of 4 cm H_2_O positive end-expiratory pressure and a fraction of inspired oxygen of 50%, demonstrated a pH of 7.24, a partial pressure of arterial oxygen (PaO_2_) of 77 mmHg, and a PaO_2_/FiO_2_ ratio of 154.

Chest computed tomography (CT) demonstrated reduced radiolucency in both lungs, accompanied by prominent, indistinct, and disorganized pulmonary markings. Multiple patchy and confluent areas of heightened attenuation were observed bilaterally, with several lesions exhibiting ground-glass opacities characterized by ill-defined borders (Figure 1B). Duplex ultrasonography of the deep veins in the lower extremities revealed no signs of thrombosis. Transthoracic echocardiography indicated a pulmonary artery systolic pressure of approximately 36 mmHg, a left ventricular ejection fraction of 70%, and preserved right ventricular systolic function. Cranial CT scanning identified hypodense regions within the occipital white matter. Cardiac color Doppler ultrasound is normal, and bilateral lower extremity deep vein B-ultrasound shows no thrombosis.

Seven hours following admission to the Respiratory Intensive Care Unit (RICU), the patient manifested acute dyspnea. Subsequent arterial blood gas analysis, conducted under conditions of 6 cmH₂O positive end-expiratory pressure (PEEP) and an inspired oxygen fraction (FiO₂) of 60%, yielded a pH of 7.24, a partial pressure of oxygen (PaO₂) of 57.2 mmHg, and a PaO₂/FiO₂ ratio of 95.3. Consequently, immediate endotracheal intubation was initiated to establish invasive mechanical ventilation. Bedside flexible bronchoscopy with bronchoalveolar lavage (BAL) was subsequently performed (Figure 1C); examination of the lavage fluid disclosed the presence of free fat droplets and lipid-laden macrophages that tested positive for Oil Red O staining (Figure 1D).

Upon admission to the respiratory intensive care unit, the patient experienced deterioration of respiratory status, necessitating escalation of care to endotracheal intubation and invasive mechanical ventilation. The therapeutic regimen included daily intravenous administration of 40 mg methylprednisolone and 2 g ceftriaxone, complemented by enteral nutritional support and intravenous fluid resuscitation. These interventions collectively enabled gradual resolution of the patient's respiratory failure. By the third day of admission, follow-up chest computed tomography revealed marked absorption of bilateral ground-glass opacities compared to baseline imaging (Figure 1E); consequently, the methylprednisolone dosage was tapered to 20 mg on day three. On day six, arterial blood gas analysis performed during pressure support ventilation with a fraction of inspired oxygen of 33% showed a pH of 7.442 and a partial pressure of arterial oxygen of 76.7 mmHg, while peripheral oxygen saturation remained stably above 96%. These results supported extubation and successful weaning from mechanical ventilation. After weaning, the patient was transitioned to heated humidified high-flow nasal cannula therapy, and methylprednisolone was discontinued. By day eight, the patient's chest tightness had fully resolved, and their clinical status had improved significantly, allowing discharge on the same day. The patient was subsequently transferred to an orthopedic facility for flap reconstruction surgery. The treatment timeline, laboratory tests, and arterial blood gas analysis results during the patient's hospitalization are shown (Table 1).

**Table 1:** Treatment timeline and laboratory tests and arterial blood gas analysis results.

Timeline	treatment measures	Laboratory and imaging studies	arterial blood gas analysis			
			Inhaled oxygen concentration (%)	PH	Partial pressure of oxygen (mmHg)	Blood oxygen saturation (%)
Day 1	Non-invasive mechanical ventilation methylprednisolone 40 mg infusion, and fluid resuscitation	Chest CT: Asymmetric multifocal patchy and large-area increased-density opacities in both lungs; White blood cell count: 10.53 × 10⁹/L; Hemoglobin: 158 g/L; CRP: 26.20 mg/L; NT-proBNP: 327 pg/mL; D-dimer: 1,020 μg/L; Troponin I, procalcitonin, liver and kidney function tests, and cardiac echocardiography were all within normal ranges	50	7.240	77	95.8
Day 2	After 7 h, the patient was transferred to tracheal intubation with invasive mechanical ventilation	Bedside tracheal lavage with Oil Red O staining revealed fat droplets in the lavage fluid; TNGS analysis of the lavage fluid showed no pathogenic bacteria; Deep vein ultrasound of both lower extremities revealed no thrombosis;	60	7.213	57.2	87.9
Day 3	Methylprednisolone dosage reduced to 20 mg via intravenous infusion	The chest CT demonstrates improvement in bilateral lung lesions compared with prior imaging.; Cranial CT scan revealed reduced white matter density in the occipital lobe	45	7.386	79.7	97
Day 4	Continued invasive mechanical ventilation	N/A	45	7.409	81	96.9
Day 5	Continued invasive mechanical ventilation	N/A	45	7.415	74.9	96
Day 6	Tracheal intubation removed; subsequent transition to non-invasive mechanical ventilation; discontinuation of intravenous methylprednisolone	N/A	33	7.442	76.7	96
Day 7	Significant clinical improvement observed, with absence of chest tightness	N/A	40	7.400	62.5	92.3
Day 8	patient transferred to the Department of Orthopedics for skin flap repair	N/A	N/A			

## Discussion

In this report, we describe a 43-year-old obese male who developed Fat Embolism Syndrome (FES) after surgery for a finger crush injury, a condition that rapidly progressed to respiratory failure. This case presents several unique features: First, while long bone fractures are the most common triggers of FES (8, 9), finger crush injuries represent an extremely rare form of atypical trauma. Although sporadic reports in the literature describe FES triggered by non-long bone fractures, such as vertebral fractures (10), the incidence of FES varies significantly depending on the affected bone and fracture type (2). Second, obesity has been established as an independent risk factor for FES in multiple studies (8, 9). This patient's profile aligns with these findings, reinforcing the role of obesity as a known predisposing factor for embolic diseases. Finally, the patient progressed from non-invasive to invasive ventilation within 12 h post-operation, underscoring the need for clinicians to remain vigilant for FES after atypical trauma. Similar rapid progression has been reported in the literature, with patients developing neurological symptoms within 12 h after multiple fractures, ultimately leading to brain death (5). Collectively, these features suggest that clinicians should maintain a high index of suspicion for FES in obese patients presenting with fulminant respiratory failure, even following minor trauma such as finger crush injuries.

In light of this uniqueness, comparing this case with existing literature further highlights its clinical significance. In terms of injury mechanism, typical FES reported in the literature primarily occurs secondary to long bone fractures of the lower extremities, particularly fractures of the femoral shaft and bilateral long bones. In contrast, the present case resulted from a finger crush injury, representing a rare etiology that broadens the known etiological spectrum of FES. With respect to diagnosis, this patient was confirmed to have fat FES through an integrative assessment comprising the history of finger crush trauma, chest CT revealing asymmetric patchy high-density opacities exhibiting a “snowstorm” pattern bilaterally, bronchoalveolar lavage (BAL) coupled with Oil Red O staining (11), and the exclusion of alternative etiologies. This confirmatory finding aligns with prior literature asserting that such an approach may serve as a rapid and specific diagnostic tool (12). Nevertheless, despite its high sensitivity, certain investigations have demonstrated that Oil Red O-positive macrophages can emerge in trauma patients lacking FES, implying that the specificity of this method necessitates comprehensive evaluation alongside established clinical diagnostic criteria, depending on BAL sampling timing, the study cohort, and the defined threshold. Furthermore, positive staining may occur in individuals with fat embolism who do not fully meet the clinical diagnostic criteria for FES; consequently, interpreting positive results requires integration with clinical manifestations for a comprehensive clinical assessment. Crucially, a negative result does not preclude the presence of fat embolism syndrome. Additionally, imaging plays a valuable role in diagnosis; for instance, MRI can detect early microscopic infarcts in the brain and indicate prognosis. The patient's cranial CT reveals low density within the occipital white matter, suggestive of embolic ischemia or cytotoxic edema caused by fat embolism, potentially indicating subclinical cerebral involvement in FES. Since the patient was intubated and required invasive mechanical ventilation, a brain MRI could not be safely performed during the acute phase. After clinical improvement, the patient exhibited no persistent neurological signs or symptoms; therefore, no follow-up MRI was conducted. For this patient, the Schonfeld was 9 points (diffuse pulmonary infiltrates on chest imaging: 5 points; hypoxemia: 3 points; tachycardia: 1 point; no petechiae, fever, or altered consciousness), which is highly suggestive of fat embolism syndrome (≥ 5 points).

Regarding the differential diagnosis, chest CT upon admission revealed diffuse bilateral patchy opacities in the lungs, suggestive of infectious lesions. CTPA was not performed due to the patient's worsening respiratory failure and immediate transfer to the ICU; consequently, pulmonary thromboembolism could not be definitively excluded, representing a study limitation. However, the likelihood of PTE is low: lower extremity venous ultrasound revealed no deep vein thrombosis, echocardiography showed normal right heart function, and no clinical manifestations of PTE were present. The patient presented without fever, cough, or sputum production; furthermore, procalcitonin levels were normal, and bronchoalveolar lavage fluid metagenomic next-generation sequencing (tNGS) results were negative, effectively excluding pulmonary infection. In this case, NT-proBNP was mildly elevated, while cardiac troponin levels and echocardiography findings were within normal ranges, and CT showed bilateral asymmetric storm-like infiltrates, ruling out pulmonary edema. Although the patient met the criteria for acute respiratory distress syndrome (ARDS) according to the Berlin definition, the respiratory failure was likely attributable to FES; therefore, ARDS is considered a clinical manifestation or complication of FES rather than an independent diagnostic entity.

In terms of treatment response, the patient improved after receiving corticosteroids and fluid therapy, which aligns with literature conclusions supporting that corticosteroids can significantly reduce the risk of FES and hypoxemia (13). Nevertheless, some systematic reviews have found insufficient evidence to support their use for prevention, and no clinical studies have definitively demonstrated that any pharmacological intervention provides unequivocal benefits for the clinical course of FES. Therefore, while this case illustrates the potential therapeutic efficacy of this drug regimen, generalizing these findings warrants caution given the rarity of this etiology. In summary, this case reinforces the utility of existing diagnostic methods and suggests that clinicians should maintain a high index of suspicion for FES triggered by atypical injuries, such as short bone fractures.

From a pathophysiological standpoint, the finger crush injury in this case likely constituted the primary source of fat emboli. Concurrently, the patient's obesity, characterized by excessive adipose reserves, facilitated the release of bone marrow or subcutaneous micro-fat globules into the circulation following trauma. This release triggered systemic inflammatory response syndrome (14), ultimately leading to progressive respiratory failure. Evidence from animal models indicates that fat embolism activates the p38 MAPK/NF-*κ*B signaling cascade, which precipitates acute lung injury and upregulates pro-inflammatory mediators, including TNF-α and IL-1β (15). Moreover, therapeutic strategies for acute lung injury prioritize suppressing the NF-*κ*B pathway and downregulating TNF-α, IL-1β, and IL-6 to mitigate inflammation (16). Clinically, managing FES focuses on prevention, early detection, and robust symptomatic support. In this instance, the combination of respiratory support, glucocorticoid administration, fluid resuscitation, and targeted symptomatic care led to clinical improvement and the resolution of acute respiratory failure secondary to FES.

At the forefront of medical research, the clinical significance of this case underscores the need for clinicians to maintain a high index of suspicion for FES in obese patients presenting with respiratory symptoms following finger crush injuries. In this case, the patient's obesity coupled with early postoperative dyspnea-an atypical presentation-provided critical clues for early identification. Early recognition and timely initiation of supportive therapy can improve prognosis. Monitoring respiratory function in obese patients warrants close surveillance after fracture surgery, particularly in the presence of dyspnea. Furthermore, early surgical fixation (within 48 h) in fracture patients can reduce the risk of FES (9). Thus, this case serves as a valuable reference for clinicians in identifying and managing FES following atypical trauma.

## Conclusion

A 43-year-old man with obesity developed rapidly progressive Fat Embolism Syndrome 12 h after surgery for a finger crush injury. Based on the history of finger crush injury, the presence of a bilateral “snowstorm” pattern characterized by asymmetric patchy high-density opacities on chest imaging, and bronchoalveolar lavage fluid analysis with oil red O staining, a diagnosis of fat embolism was confirmed. The patient was successfully weaned from mechanical ventilation followingrespiratory support, glucocorticoid therapy, and general supportive care. This case underscores that in cases of atypical trauma, particularly among obese patients, the onset of early respiratory symptoms should warrant a high index of suspicion for Fat Embolism Syndrome, thereby necessitating immediate diagnostic and therapeutic intervention.

## Data Availability

The original contributions presented in the study are included in the article/Supplementary Material, further inquiries can be directed to the corresponding author.
